# Coping Strategies of Parents of Children with Autism Spectrum Disorders after Psychoeducation

**DOI:** 10.1192/j.eurpsy.2023.845

**Published:** 2023-07-19

**Authors:** M. Ivanov, O. Bogacheva, A. Koval-Zaytsev, E. Balakireva

**Affiliations:** 1 Department of Child Psychiatry, Federal State Budget Scientific Institution “Mental Health Research Centre”, Moscow, Russian Federation

## Abstract

**Introduction:**

Not only children with autism spectrum disorders (ASD) need specialized care, but the whole family as a whole. The family is seen as an important resource in the treatment and rehabilitation process.

**Objectives:**

To identify coping strategies for parents of children with ASD after psychoeducation.

**Methods:**

The study involved 75 families (75 mothers and 68 fathers aged 27 to 38) raising children with ASD (age range from 3 to 5 years). All children were diagnosed under subheading F84 “Pervasive developmental disorders” according to ICD-10 (F84.01; F84.02; F84.11; F84.12). Diagnosis period for a child: from 6 months to 1 year.

The main instrument was “Ways of Coping Checklist” (R. Lazarus & S. Folkman) (in Russian adaptation by L.I. Wasserman et al.).

Psychoeducational work was carried out with all parents, including:

- attitude and acceptance of the child’s illness;

- awareness of the disease and ways of helping a child with ASD;

- compliance with the recommendations of specialists working with the child (psychiatrist, psychologist, speech therapist, social pedagogue);

- skills of interaction with the child in the conditions of the house and society;

- emotional experiences of parents (anxiety due to insufficient information about the child’s illness);

- emotional acceptance of the child.

**Results:**

After completing the psychoeducational program, the parents’ scores on the “positive reappraisal” scale increased, which may indicate that parents, in spite of everything, are looking for positive aspects in the situation of raising a child with ASD and focus their attention on their own personal growth. Thus, mothers of children often undergo training in programs for working with children with ASD, begin to conduct educational webinars, blogs, and share their experience in solving problems with other parents. There is also an increase in scores on the “confrontation” scale, as well as a decrease on the “distancing” scale.

**Conclusions:**

Conducting psychoeducation with the parents of underage patients allows us to come closer to solving one of the main issues of psychosocial rehabilitation - the socialization of a child with ASD and the whole family as a whole.

**Disclosure of Interest:**

None Declared

